# The effect of combining vibratory platform and unstable footwear on static balance in active young people

**DOI:** 10.1038/s41598-022-07926-6

**Published:** 2022-03-10

**Authors:** C. Varangot-Reille, P. Salvador-Coloma, G. Biviá-Roig, P. Múzquiz-Barberá, J. F. Lisón

**Affiliations:** 1 Department of Nursing and Physiotherapy, Faculty of Health Sciences, University CEU-Cardenal Herrera, CEU Universities, C/Ramón Y Cajal S/N, 46115 Alfara del Patriarca, Valencia, Spain; 2 Department of Biomedical Sciences, Faculty of Health Sciences, University CEU-Cardenal Herrera, CEU Universities, Valencia, Spain; 3 Centre of Physiopathology of Obesity and Nutrition (CIBERobn), CB06/03 Carlos III Health Institute, Valencia, Spain

**Keywords:** Orthopaedics, Rehabilitation

## Abstract

Vibratory platforms (VPs) and unstable footwear (UF) have both shown benefits on balance in some populations. However, there is no evidence about the combined effects of using UF while training on an VP in healthy and physically active young people. We aimed to evaluate the effects of wearing unstable footwear (UF) while training on a whole-body VP on balance in healthy, physically active young people. 23 participants were randomized into groups assigned UF (*n* = 11) or stable footwear (SF; *n* = 12). Both groups followed the same training program on an VP with the assigned footwear type twice a week for 12 weeks. The training consisted of performing 8 isometric exercises for progressively longer periods and higher oscillation amplitudes (15–60 s, 1–3 mm), at a fixed vibration frequency (20 Hz). The main outcomes were the antero-posterior and medio-lateral velocities of the center of pressure (COP) recorded using a plantar pressure corridor at baseline, post-treatment and 1-month follow-up. We found a statistically significant difference in the antero-posterior velocity during the monopodal test in the UF group between the different time-points (χ^2^(2) = 13.282, p = 0.001). Mediolateral COP velocity ranking during the bipodal test was lower for UF than for SF group (U = 19.50, z = − 2.86, p = 0.003) at follow-up. The traditional vibratory platform training does not seem to be effective to improve static balance in physically active young people, however, adding UF provided slightly greater effect.

## Introduction

The use of vibratory platforms (VPs) to improve physical fitness has increased in recent years. Several meta-analysis have concluded that whole body vibration (WBV) is relatively effective in improving balance in different populations, such as older adults^1^ or patients with knee osteoarthritis^2^.

Several mechanisms have been hypothesized to explain the improved balance following the application of WBV. Tonic vibration reflex is considered the most commonly accepted theory by activating muscle spindles, it enhances the excitatory drive reflex of the α-motor neurons via group Ia afferent fibers^3^. Additionally, WBV inhibits spinal reflexes and prevents the occurrence of reflex-mediated joint oscillations that can compromise postural control mechanisms and shift movement control towards supraspinal levels^4^. Increased neuromuscular activation in response to WBV can lead to enhanced co-contraction of lower limb muscles, which in turn can increase joint stiffness and contribute to improved balance^5^. Several authors have found that traditional VP training may not be effective to improve static balance in active young participants^6,7^, however, the heterogeneity in WBV parameters, the combination of VP with unstable platforms and the possible synergistic effects are still understudied.

Training on unstable surfaces is another accepted method to improve balance and postural control. In this context, unstable footwear (UF) with curved soles has been used for improving strength and balance^8,9^; their rounded soles induce instability in the antero–posterior and medio-lateral directions^10^ and, accordingly, studies have found that standing in UF can immediately increase postural sway and lower limb muscle activities compared to flat shoes and barefoot^8,11^. Continuous impulse to proprioceptive receptors -especially those of the lower extremities- and enhanced muscular activity can be considered probable mechanisms behind the beneficial effects of UF^9^. In this sense, a recent study revealed a significant increase in the electromyographic activity of the trunk muscles (erector spinae, rectus abdominis, internus obliquus, and externus obliquus) and an improvement in disability in chronic low back pain patients following the use of UF^12^.

In view of all these findings, it is of interest to evaluate the effect of training on a VP while using UF as a means to improve balance. In this sense, some authors have tried to enhance the improvements in balance produced by using VPs by studying the possible benefits of adding unstable surfaces to training sessions using VPs and found promising results^13–15^. It is reasonable to speculate that lateral accelerations may introduce more instability when standing using UF with curved soles in the medio-lateral and antero-posterior directions. Specifically, combining different positions on an oscillating VP (in line [X-axis] or perpendicular [Y-axis] to the fulcrum) while wearing UF could generate greater medio-lateral and antero-posterior instability.

Static balance, specifically the center of pressure (COP) velocity, has been shown to be a risk factor for noncontact anterior cruciate ligament injury in handball and basketball players^16^. Similarly, McGuine et al. found that worse static balance predicted a higher risk of ankle sprain in a sample of young basketball players^17^. In fact, they found a statistically significant difference in ankle sprain injuries rate between the group with the greater and worst static balance, with 0.40/1000 exposures and 2.68/1000 exposures respectively^17^.

To the best of our knowledge, no studies have yet evaluated the combined effects of using UF while training on an oscillating VP in healthy and physically active young people. We hypothesized that any improvement in static balance would be significantly greater after training with UF on an oscillating VP compared to the same training while using stable footwear.

## Methods

We conducted a preliminary randomized controlled trial with a parallel design. The study was approved by the local Ethics Committee at the CEU Cardenal Herrera University (Valencia, Spain). The principles of the Declaration of Helsinki were respected at all times. The study was structured according to the indications set out in the Consort guidelines. A protocol was previously registered in an international online database (NCT04011423; 08/07/2019).

### Participants

We used the following inclusion criteria to recruit participants into the study: individuals who were (1) healthy; (2) aged between 18 and 40; (3) accustomed to a moderate or high level of physical activity according to the *International Physical Activity Questionnaire Short Form* (IPAQ-SF); (4) had not undergone VP training in the year prior; and (5) had not used UF in the year prior. Individuals with (1) acute low back pain; (2) decompensated coronary diseases; (3) musculoskeletal injuries; (4) any other pathology that contraindicated the use of an VP or UF were excluded from the study.

Students from the CEU Cardenal Herrera University were recruited between March 2018 and June 2019. All potentially eligible individuals interested in participating were invited to an interview with the researcher responsible for the recruitment. Those who met the inclusion criteria were given a second appointment during which they signed their informed consent to participation and their baseline variables were measured. After the pre-intervention evaluation, participants were randomly assigned either to the stable footwear (SF) study group or the UF group.

Before the start of the program, researcher 1, who was not involved in the selection or inclusion of participants, prepared numbered, sealed, opaque envelopes for the volunteers containing the allocation groups. Investigator 2 used a simple randomization algorithm to generate a random number sequence with a 1:1 allocation rate using a computerized random number generator. The allocation sequence for each group was hidden from all the research staff involved in processing the data throughout the duration of the study and the data analysis. Because of the nature of the intervention, the volunteers were not blinded to the intervention.

### Intervention

**Figure 1:**
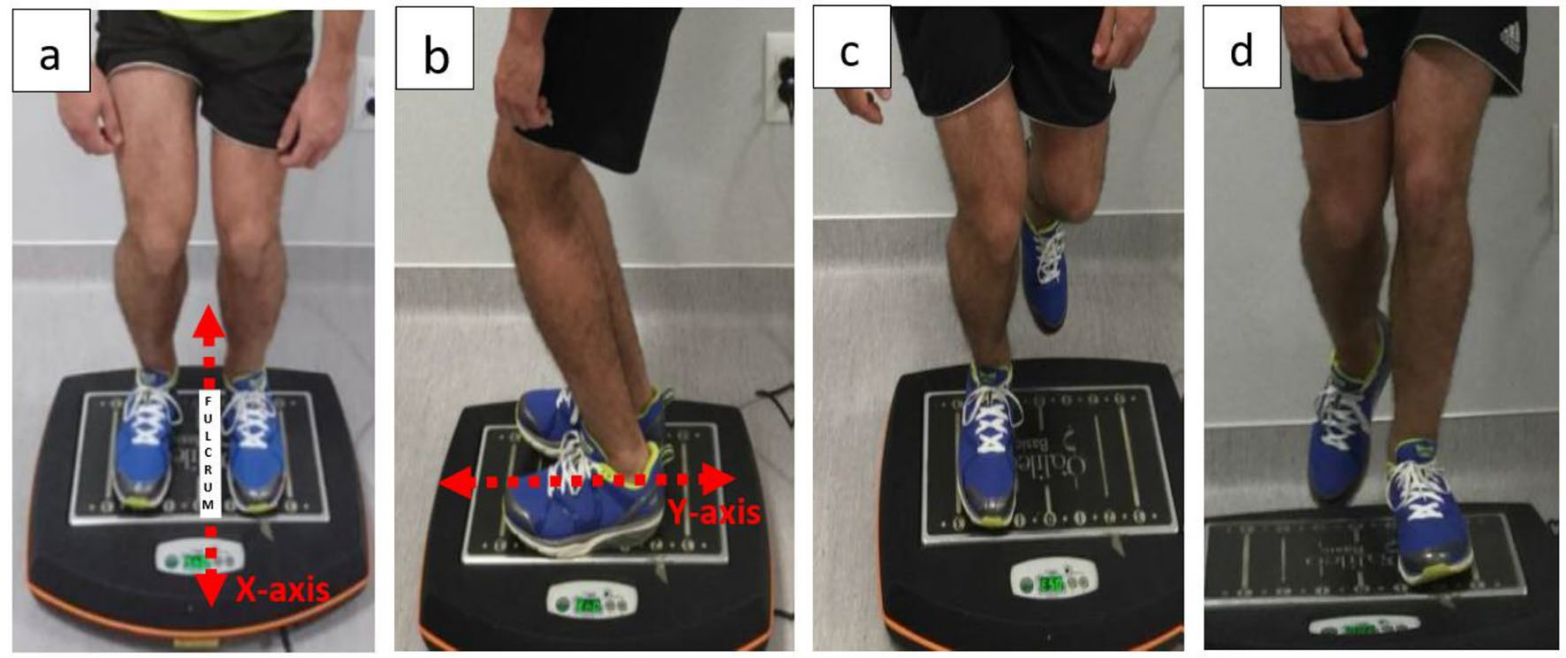
Vibratory platform positions—(**a**) X-axis bipodal; (**b**) Y-axis bipodal; (**c**) X-axis monopodal right leg; (**d**) X-axis monopodal left leg.

Both groups followed the same 12-week training program with training sessions twice a week on non-consecutive days for a total of 24 sessions. Each training session began with a 6-min warm-up consisting of walking on a treadmill with the assigned SF or UF. The training on the oscillating VP consisted of performing 8 isometric exercises [(1) bipodal X-axis support with open eyes; (2) bipodal X-axis support with closed eyes; (3) bipodal Y-axis support with open eyes; (4) bipodal Y-axis support with closed eyes; (5) right foot monopodal support with open eyes; (6) right foot monopodal support with closed eyes; (7) left foot monopodal support with open eyes; (8) left foot monopodal support with closed eyes (Fig. 1), on the oscillating VP at a fixed vibration frequency (20 Hz) and with progressively increasing oscillation lengths and amplitudes (15–60 s and 1–3 mm). The exercises were always performed in the same order (Supplementary content 1).

Two series of the 8 exercises were performed with one minute of recovery time allowed between each exercise. Supplementary content 2 shows the progression of the training load (duration and oscillation amplitude) over the 24 sessions. The knee flexion–extension angle (60° in flexion) was controlled using a goniometer during all of the exercises^18^. All the volunteers continued with their usual physical activities throughout the study period. The training was carried out on a 470 × 270 mm Galileo Basic® oscillating VP^19^ (Novotec Medical GmbH, Pforzheim, Germany) which weighed 35.5 kg and had a maximum load of 120 kg (230 V AC electrical system, 50–60 Hz and 400 VA). This model generates oscillating alternative vibrations around a fulcrum with amplitudes of 0–3.9 mm and frequencies of 5–30 Hz.

**Figure 2:**
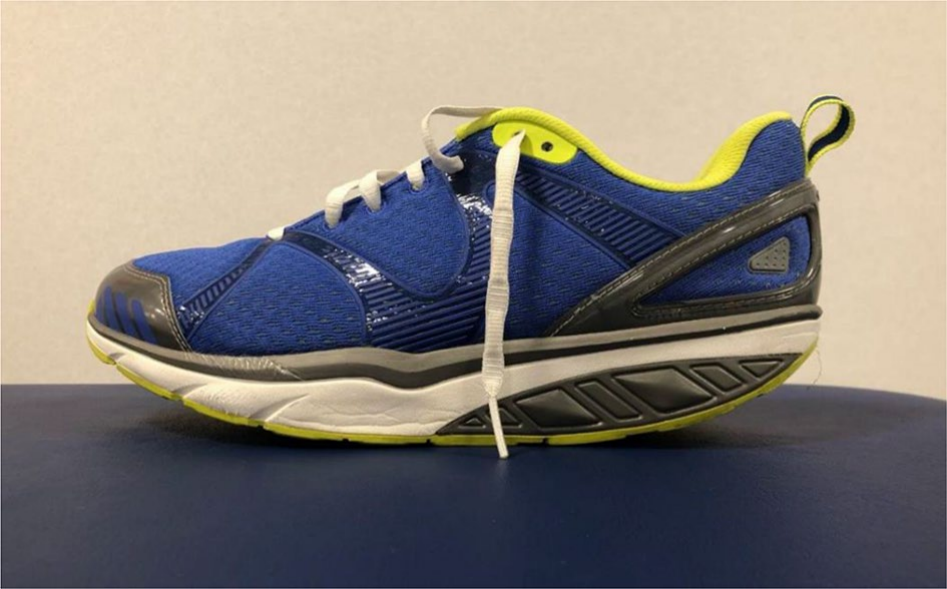
Side view of the unstable footwear.

MBT brand UF were used in this study. These shoes are characterized by their curved soles that create instability in the antero-posterior and medio-lateral direction and their flexible heel that makes the base unstable (Fig. 2). The control group SF used Jack Smith brand shoes with a flat sole.

### Study variables

The study variables were the antero-posterior (AP) and medio-lateral (ML) COP velocities which were recorded on a Win-Track plantar pressure platform (Medicapteurs Co., Balma, France). The dimensions of this device are 1610 mm × 652 mm × 30 mm (length/width/height); the thickness of the platform is 9 mm composed of 12,288 resistive type sensors. This instrument measures the COP, with a frequency of 40 Hz in the chosen position; the plantar pressure platform data were transformed into COP measurements using calibration equations. The antero-posterior and medio-lateral components of the COP were extracted. All data time series were filtered and numerically differentiated to produce COP velocity measures.

The process was identical between groups. In each trial, the average velocities (antero-posterior and medio-lateral) of the COP were used for analysis. COP velocity has been shown to be reliable between sessions (R = 0.84)^20^. The static positions used to measure the COP velocities were (1) static bipodal support with open eyes and (2) static monopodal support on the dominant foot with open eyes.

We recorded the COP velocity in both the control and intervention groups while they were wearing SF and standing on the plantar pressure platform. The volunteers were instructed to hold and maintain each position with the least possible body movement for 30 s, three times for each trial. The mean of the averaged COP velocities (antero-posterior and medio-lateral) of these three repetitions was subsequently calculated in our statistical analysis. We recorded the study variables before the intervention, at the end of the intervention and four weeks after the end of the last training session by a trained physiotherapist who was blinded to the group assignment. Participants were also asked about the presence or occurrence of any adverse effects during or after each training session using the oscillating VP.

The IPAQ-SF was used to categorize the level of physical activity of each participant. This questionnaire comprises a series of items that quantify the physical activity performed by an individual during the previous week in order to categorize the volunteer’s normal physical activity levels as ‘light’ (category 1), ‘moderate’ (category 2), or ‘high’ (category 3). This questionnaire has been previously validated to assess activity levels in young adults^21^.

### Statistical analysis and study size

The sample size was calculated based on the results from a pilot study with 16 participants we previously undertook which indicated an effect size of f = 0.34 for the medio-lateral COP velocity. Considering this effect size, the desired power of 80%, and setting the α value at 0.05, we used G*Power software^22^ to estimate that an overall sample size of 20 participants would be required in this current study. Accounting for possible losses of 15%, we established the final desired sample size as a total of 23 participants.

We used the Shapiro–Wilk test to assess if the outcomes were normally distributed. All primary outcomes in our study were not normally distributed, there were expressed as median ± interquartile range (IQR). Baseline characteristics were expressed as mean ± standard deviation (SD) or median ± IQR, depending on whether the data were normally distributed or not.

We tested within-group effect using Friedman Test over time. The values p < 0.05 were considered statistically significant. If there was any significant difference, post-hoc analyses were executed running Wilcoxon-sign ranked tests between the different time points. We tested between-group differences using Mann–Whitney’s U tests at each time points.

Statistical analysis was done using SPSS software, version 27.0 (IBM Corp., Armonk, N.Y., USA). All statistical analyses were applied with intention-to-treat.

## Results

**Figure 3:**
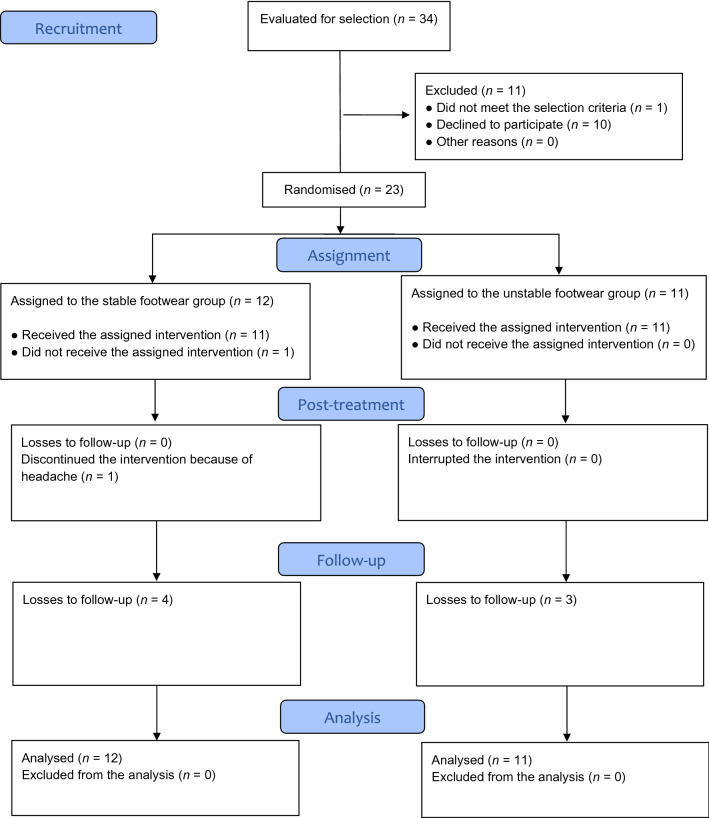
Flow chart of the study participants.

A total of 34 volunteers were assessed for eligibility; 11 of these were not assigned for randomization because they did not meet the inclusion criteria (1 had acute low back pain and 10 declined to participate). Thus, 23 participants were evaluated at baseline and were randomized into the SF (*n* = 12) or UF (*n* = 11) groups. A total of 22 participants received the intervention, 21 were evaluated at the end of the treatment and 15 of these were evaluated at follow-up. The only adverse effect observed in this study was a participant from the stable footwear group reporting an episode of headache after the first training session (*n* = 1). Table 1 shows the baseline characteristics of the trial participants, no group differences were observed at baseline. Figure 3 shows how they progressed through the trial.

**Table 1:** Participant characteristics at baseline^a^.

Characteristics	Stable footwear group (n = 12)	Unstable footwear group (n = 11)	P-value
Gender (women/men)	7/5	6/5	0.743
Age (years)	22.9 (2.4)^b^	22.4 (2.4)^b^	0.585
Weight (kg)	65.7 (11.3)^b^	64.9 (12.2)^b^	0.913
Height (cm)	170.1 (7.3)^b^	169.6 (7.7)^b^	0.493
BMI (kg/m^2^)	22.2 (21.9–24.1)^c^	20.5 (19.1–24.6)^c^	0.151
IPAQ category	2.0 (2.0–3.0)^c^	3.0 (2.0–3.0)^c^	0.566

The data shown are the mean (standard deviation (*SD*)) or median (interquartile range (IQR)).

BMI = body mass index; IPAQ = International Physical Activity Questionnaire.

^a^No group differences were observed.

^b^Mean (standard deviation (*SD*)).

^c^Median (interquartile range).

Table 2 shows the results at baseline, post-treatment and at a 4-weeks follow-up after the end of the intervention.

**Table 2:** Outcome measures at pre-treatment, post-treatment, and follow-up.

Test condition	Variables	Group							Time	
		Pre-treatment			Post-treatment		Follow-up		p-valor	
		SF	UF	p-valor	UF	p-valor	UF	p-valor	SF	UF
Bipodal	AP COP velocity (mm/s)	2.7 (2.2–3.4)	2.9 (2.5–3.4)	0.734	3.0 (2.9–3.1)	0.347	2.8 (2.4–4.2)	0.413	0.741	0.903
	ML COP velocity (mm/s)	2.7 (2.2–4.2)	2.4 (2.2–3.3)	0.228	2.4 (1.7–3.1)	0.134	2.0 (1.6–2.4)	0.003^a^	0.717	0.704
Unipodal	AP COP velocity (mm/s)	8.3 (7.7–10.4)	10.2 (7.9–11.2)	0.356	7.6 (7.1–9.8)	0.695	8.3 (7.5–9.6)	0.740	0.497	0.001^a^
	ML COP velocity (mm/s)	8.6 (7.8–10.9)	9.1 (8.5–12.9)	0.340	8.2 (6.5–9.6)	0.880	8.1 (7.1–9.5)	0.525	0.452	0.070

Data are expressed as median (IQR). Time effect was analyzed with Friedman test. Between-group effect was analyzed using Mann–Whitney’s U tests at each time points.

*AP*: Anteroposterior; *COP*: Centre of Pressure; *ML*: Mediolateral; *SF*: Stable footwear; *UF*: Unstable footwear.

^a^Statistically significant difference (p < 0.05).

We found a statistically significant difference in the antero-posterior velocity in the UF group during the monopodal test between the different time-points (χ^2^(2) = 13.282, *p* = 0.001). There was statistically significant reduction in the antero-posterior velocity during the monopodal test between baseline and post-intervention (Z = -2.313, p = 0.021) and between baseline and follow-up (Z = -2.936, p = 0.003). However, there was no further improvement between post-intervention and follow-up (Z = -0.734, p = 0.463). Table 3 shows the *post-hoc* analysis. We did not find a statistically significant difference in the antero-posterior velocity in the SF group during the monopodal test between the different time-points (χ^2^(2) = 1.590, *p* = 0.452). Neither the stable footwear group, nor the unstable footwear group had significant improvement in static balance in the bipodal position.

**Table 3:** Post-hoc analysis between time-points.

Test condition	Variables	Group	Post—Pre	Follow-up—Pre	Follow-up—Post
			p-valor	p-valor	p-valor
Unipodal	AP COP velocity (mm/s)	UF	p = 0.021^a^	p = 0.003^a^	p = 0.463

Post-hoc analyses were executed running Wilcoxon-sign ranked tests between the different time points.

*AP*: Anteroposterior; *COP*: Centre of Pressure; *I*: Impact effect size; *UF*: Unstable footwear.

^a^Statistically significant difference (p < 0.05).

A Mann–Whitney test indicated that the mediolateral COP velocity ranking during the bipodal test was lower for unstable footwear (Median (Mdn) = 2.0) than for stable footwear group (Mdn = 3.7) (U (N_UF_ = 11, N_SF_ = 12) = 19,50, z = − 2,86, p = 0.003) at follow-up. However, no other outcome at any time showed between-group significant differences.

## Discussion

We aimed to evaluate the effect of a training of 12 weeks combining vibratory platform with unstable shoes on static balance. We found that the combination of unstable footwear and vibratory platform had poor effects on static balance. We found that this combination had a statistically significant effect on the anteroposterior velocity of COP over time, however, without between-group difference neither at follow-up, nor post-treatment. We found a between-group statistically significant difference on the mediolateral velocity of COP at follow-up, but that was the only difference. We wanted to highlight that the traditional training on vibratory platform with stable footwear did not improve static balance.

To date, only one study evaluating the effect of training on an oscillating VP with UF has been published^15^. In this study, Sobhani et al. used a VP type (Fitvibe®WBV unit) whose whole plate moves up and down (vertically VP). Interestingly, there are other types of VPs which deliver reciprocating vertical displacements on the left and right side of a fulcrum, increasing the lateral accelerations (oscillating VP)^23^. Contrary to our results, Sobhani et al. reported significant improvements in balance at one month follow-up after the 12-week training period and concluded that combining WBV with UF can be proposed as a beneficial method with relatively long-term effects to improve balance measures in older people^15^. However, this study was conducted in a geriatric population and did not use a precise balance assessment (Fullerton Advanced Balance scale)^15^. Marin and Hazell^14^ examined the effects of using an unstable surface during WBV exercise on leg and trunk muscle activity during a static semi-squat in healthy university students. Compared to a stable surface, WBV exercising on a wobble board led to significantly higher electromyographic activity in the calf, quadriceps, and lower back muscles. Sierra-Guzmán et al.^13^ evaluated the effects of a 6-week WBV training program, performed on a soft, unstable surface, on peak torque, reaction time and the electrical activity of ankle muscles in recreational athletes with chronic ankle instability. They reported a significant improvement in reaction times of the peroneus longus and tibialis anterior muscles, whereas no significant changes were found in the control group. However, despite a theoretical possible influence of the VP and UF combination on the lower limb neuromuscular activity, we found that it does not seem to be greatly reflected on the postural control of physically active young people.

We also found that the use of VP only did not improve static balance in active young people. This is in accordance with the results of several studies who also did not find this training modality to be effective to improve balance in active young people^6,7^. Actually, Hiroshige et al. found that an 8-weeks training of VP was not effective to improve static balance in young people, but it was in elderly people^24^. Other studies found similar results in elderly or patients with any kind of pathology^25,26^. It could explain why we did not find almost any improvement but Sobhani et al. did find it with elderly people^15^. Traditional VP training does not seem to be effective to improve static balance in young people and we do not recommend its use with healthy and physically active young people with the aim to improve static balance. Future investigations studying the VP and UF combination on balance should focus on elderly population or functionally impaired populations.

The slightly higher improvement of the unstable footwear group might be attributed to the use of unstable footwear. A recent systematic review by Papalia et al.^9^ concluded that wearing UF in a static standing position displaces the COP to a more posterior position which, in turn, increases lumbar erector spinae and intrinsic ankle musculature activity, thus improving balance. Likewise, Landry et al.^11^ concluded that the use of UF improves neuromuscular coordination and activation at a more distal level, allowing better support strategies to be sustained and thus, enhancing balance at the level of the ankle. Future studies should compare a training combining VP and UF with only UF in order to discriminate if we found a cumulative and/or synergic positive effect on the mediolateral velocity during the unipodal test or just the effect of UF training on static balance.

In our study we did not find any significant improvements in the antero-posterior or medio-lateral COP velocities in the bipodal test with the SF or UF. Bipodal support with open eyes is a fairly common position in everyday life and, given that our participants were young and physically active, their margin for improvement was likely very small for simple tasks^27^, perhaps explaining these findings. Indeed, Marcolin et al. found that in young and healthy gymnasts, experience levels did not influence postural balance control for simple tasks^28^. Because this was a controlled position that was not demanding for the population we included, the ceiling effect have certainly been reached with relative ease, meaning that any improvements have been small or null. Because older individuals have more balance impairments, the balance improvements found by Sobhani et al. might have been due to a greater margin of improvement in older participants^15,29^.

Here, we analyzed the movement of the COP to assess balance, which is now one of the most common methods for measuring postural control^30^. Movement of the COP measures postural balancing and corresponds to the small adjustments made by the body to maintain mono- or bipodal balance. The study variables we examined here were the antero-posterior and medio-lateral COP velocity as a way to measure the effectiveness of the postural control system neuromuscular activity adjustments required to maintain balance^30^. A recent review concluded that COP velocity is the most reliable of the different variables corresponding to COP movement^31^.

The main limitation of this study was the small sample size of the cohort we analyzed; even though we based our sample size calculations on our previous pilot study, there were 8 losses to follow-up, which meant that our statistical analysis was underpowered. Another limitation was that, because of the nature of the study, we were unable to blind the participants to the intervention. Finally, we did not record the level of physical activity the participants engaged in during the intervention period, and this could have acted as a confounding factor. Nonetheless, the random assignment of the participants to each group would have minimized this possible effect if it occurred.

In conclusion, this study showed that the traditional VP training does not seem to be an effective training to improve static balance in a healthy and physically active young population, however, the use of UF in combination with the oscillating VP could have benefits on a complex static balance task. Future studies are needed to assess whether the benefits come from a synergistic effect of UF and VP or from UF alone.

## Supplementary Information

Supplementary Information.
