# Supplementary material for: The effect of combining vibratory platform and unstable footwear on static balance in active young people
Source: Sci Rep. 2022 Mar 10;12:3931. doi: 10.1038/s41598-022-07926-6 (PMC8913682; doi:10.1038/s41598-022-07926-6)
Supplement: Supplementary file 1 — Supplementary Information. [file 41598_2022_7926_MOESM1_ESM.pdf]

The Effect of Combining Vibratory Platform and Unstable Footwear on Static Balance in Active Young People

Varangot-Reille C[1*]; Salvador-Coloma P[1]; Biviá-Roig G[1]; Múzquiz-Barberá P[1]; and Lisón JF[2,3]

1. Department of Nursing and Physiotherapy, Faculty of Health Sciences, University CEU-Cardenal Herrera, CEU Universities, Valencia, Spain
2. Department of Biomedical Sciences, Faculty of Health Sciences, University CEU-Cardenal Herrera, CEU Universities, Valencia, Spain
3. Centre of Physiopathology of Obesity and Nutrition (CIBERobn), CB06/03 Carlos III Health Institute,Valencia, Spain.

***Corresponding author: Clovis Varangot-Reille**

0034961369000; C/Ramón y Cajal s/n, 46115 Alfara del Patriarca, Valencia (Spain) ;: clovis.varangotreille@gmail.com

Supplementary Content 1. Order of positions during the training on the vibrating platform.

X-axis bipodal with eyes open
1-minute break
X-axis bipodal with eyes open
1-minute break
X-axis bipodal with eyes closed
1-minute break
X-axis bipodal with eyes closed
1-minute break
Y-axis bipodal with eyes open
1-minute break
Y-axis bipodal with eyes open
1-minute break
Y-axis bipodal with eyes closed
1-minute break
Y-axis bipodal with eyes closed
1-minute break
X-axis monopodal with right leg with eyes open
1-minute break
X-axis monopodal with right leg with eyes open
1-minute break
X-axis monopodal with right leg with eyes closed
1-minute break
X-axis monopodal with right leg with eyes closed
1-minute break
X-axis monopodal with left leg with eyes open
1-minute break
X-axis monopodal with left leg with eyes open
1-minute break
X-axis monopodal with left leg with eyes closed
1-minute break
X-axis monopodal with left leg with eyes closed

Supplementary content 2. Progression of the load (time and vibration amplitude) used in the different training sessions.

	X-axis bipodal with eyes open/closed	Y-axis bipodal with eyes open/closed	X-axis monopodal with left/right leg and eyes open	X-axis monopodal with left/right leg and eyes closed
Session 1	15 s – 2 mm	15 s – 2 mm	15 s – 1 mm	15 s – 1 mm
Session 2	15 s – 2 mm	15 s – 2 mm	15 s – 1 mm	15 s – 1 mm
Session 3	15 s – 2 mm	15 s – 2 mm	15 s – 1 mm	15 s – 1 mm
Session 4	15 s – 2 mm	15 s – 2 mm	15 s – 1 mm	15 s – 1 mm
Session 5	25 s – 2 mm	25 s – 2 mm	15 s – 1 mm	15 s – 1 mm
Session 6	25 s – 2 mm	25 s – 2 mm	15 s – 1 mm	15 s – 1 mm
Session 7	25 s – 2 mm	25 s – 2 mm	15 s – 2 mm	15 s – 1 mm
Session 8	25 s – 2 mm	25 s – 2 mm	15 s – 2 mm	15 s – 2 mm
Session 9	30 s – 2 mm	25 s – 2 mm	15 s – 2 mm	15 s – 2 mm
Session 10	30 s – 2 mm	25 s – 2 mm	25 s – 1 mm	15 s – 2 mm
Session 11	30 s – 2 mm	25 s – 2 mm	25 s – 1 mm	15 s – 2 mm
Session 12	30 s – 2 mm	25 s – 2 mm	25 s – 1 mm	15 s – 2 mm
Session 13	38 s – 2 mm	25 s – 2 mm	25 s – 2 mm	15 s – 3 mm
Session 14	38 s – 2 mm	25 s – 2 mm	25 s – 2 mm	15 s – 3 mm
Session 15	38 s – 2 mm	25 s – 2 mm	25 s – 2 mm	15 s – 3 mm
Session 16	45 s – 2 mm	25 s – 2 mm	35 s – 1 mm	25 s – 1 mm
Session 17	45 s – 2 mm	25 s – 2 mm	35 s – 1 mm	25 s – 1 mm
Session 18	45 s – 2 mm	25 s – 2 mm	35 s – 1 mm	25 s – 1 mm
Session 19	52 s – 2 mm	25 s – 2 mm	35 s – 2 mm	25 s – 2 mm
Session 20	52 s – 2 mm	25 s – 2 mm	35 s – 2 mm	25 s – 2 mm
Session 21	52 s – 2 mm	25 s – 2 mm	35 s – 2 mm	25 s – 2 mm
Session 22	60 s – 2 mm	25 s – 2 mm	45 s – 1 mm	25 s – 3 mm
Session 23	60 s – 2 mm	25 s – 2 mm	45 s – 1 mm	25 s – 3 mm
Session 24	60 s – 2 mm	25 s – 2 mm	45 s – 1 mm	25 s – 3 mm
